# Author Correction: Deep-learning density functional theory Hamiltonian for efficient ab initio electronic-structure calculation

**DOI:** 10.1038/s43588-024-00723-3

**Published:** 2024-10-23

**Authors:** He Li, Zun Wang, Nianlong Zou, Meng Ye, Runzhang Xu, Xiaoxun Gong, Wenhui Duan, Yong Xu

**Affiliations:** 1 State Key Laboratory of Low Dimensional Quantum Physics and Department of Physics, Tsinghua University, Beijing, China; 2 Institute for Advanced Study, Tsinghua University, Beijing, China; 3 School of Physics, Peking University, Beijing, China; 4 Frontier Science Center for Quantum Information, Beijing, China; 5 Beijing Academy of Quantum Information Sciences, Beijing, China; 6 RIKEN Center for Emergent Matter Science (CEMS), Wako, Saitama Japan

**Keywords:** Electronic properties and materials, Electronic structure, Computational methods

Correction to: *Nature Computational Science* 10.1038/s43588-022-00265-6, published online 23 June 2022.

This paper was originally published under a standard Springer Nature license (© The Author(s), under exclusive licence to Springer Nature America, Inc.). It is now available as an open-access paper under a Creative Commons Attribution 4.0 International license, © The Author(s). The error has been corrected in the HTML and PDF versions of the article.

