# Supplementary material for: Deep-learning density functional theory Hamiltonian for efficient ab initio electronic-structure calculation
Source: Nat Comput Sci. 2022 Jun 23;2(6):367–77. doi: 10.1038/s43588-022-00265-6 (PMC11499279; doi:10.1038/s43588-022-00265-6)
Supplement: Supplementary file 1 — Details of computational methods and results, Supplementary Figs. 1–21 and Tables 1–5. [file 43588_2022_265_MOESM1_ESM.pdf]

nature portfolio

https://doi.org/10.1038/s43588-022-00265-6

Supplementary information

Deep-learning density functional theory Hamiltonian for efficient ab initio electronic-structure calculation

In the format provided by the
authors and unedited

This PDF file includes:
Supplementary Sections 1 to 7
Supplementary Figures 1 to 21
Supplementary Tables 1 to 5

CONTENTS

Supplementary Section 1. Computational cost comparison 2

Supplementary Section 2. Summary of the dataset 2

Supplementary Section 3. Local coordinate and gauge transformation 3
Supplementary Section 3.1. Gauge transformation 3
Supplementary Section 3.2. Definition of local coordinate 3
Supplementary Section 3.3. Importance of local coordinate message passing (LCMP) Layer 4
Supplementary Section 3.4. Ablation studies on the LCMP layer 5

Supplementary Section 4. Detailed results of example materials studies 7
Supplementary Section 4.1. Monolayer graphene 7
Supplementary Section 4.2. Carbon nanotubes (CNTs) 8
Supplementary Section 4.3. Monolayer MoS_2 9
Supplementary Section 4.4. Twisted bilayer graphene (TBG) 9
Supplementary Section 4.5. Twisted bilayer bismuthene (TBB) 10
Supplementary Section 4.6. Bulk silicon 11
Supplementary Section 4.7. Solid structures with multiple crystalline phases 12

Supplementary Section 5. Principal component analysis (PCA) 13
Supplementary Section 5.1. PCA for nanotubes 13
Supplementary Section 5.2. PCA for twisted bilayers 14

Supplementary Section 6. Comparison with other methods 16
Supplementary Section 6.1. Comparison with the kernel ridge regression method 16
Supplementary Section 6.2. Comparison with SchNOrb and PhiSNet 16

Supplementary Section 7. Comparison of theoretical methods for twisted materials 17

References 17

Supplementary Section 1. COMPUTATIONAL COST COMPARISON

We compare the computational cost to construct density functional theory (DFT) Hamiltonian matrices by DFT and DeepH for flat supercells and curved nanotubes of graphene and MoS_2 (Supplementary Table 1). For monolayer graphene (MoS_2), we train 169 (4) message passing neural network (MPNN) models, which share the same neural network architecture but are trained for different orbital pairs. Note that decreasing the number of MPNN models can improve the computational efficiency, which usually has minor influence on prediction accuracy as demonstrated for MoS_2. Compared to DFT, DeepH can significantly save computation resource, especially for large-scale material systems. DFT calculations of graphene 6×6 supercell, (25, 0) carbon nanotube (CNT), MoS_2 5×5 supercell, and (50, 0) MoS_2 nanotube and all the DeepH calculations are done by 64 cores (one compute node equipped with 2 AMD EPYC 7542 CPUs). DFT calculations of graphene 36×36 supercell and MoS_2 25×25 supercell are done by 180 cores (5 compute nodes each equipped with 2 Intel Xeon 6240 CPUs). DFT calculations for graphene 48×48 supercell and MoS_2 35×35 supercell are done by 360 cores (10 compute nodes each equipped with 2 Intel Xeon 6240 CPUs). We use OpenMX software package to perform all the DFT self-consistent field calculations for computational cost comparison.

Supplementary Table 1. Computational cost comparison between DFT and DeepH to construct DFT Hamiltonian matrices for flat supercells and curved nanotubes of graphene and MoS_2. The unit of computational time is per second per CPU core.

Material structure	Number of MPNN models	Number of atoms	DFT time (CPU core seconds)	DeepH time (CPU core seconds)
Graphene 6×6 supercell	169	72	4,672	3,520
Graphene 36×36 supercell	169	2,592	2,311,200	67,648
Graphene 48×48 supercell	169	4,608	20,072,880	120,448
(25, 0) CNT	169	100	15,616	4,992
MoS_2 5×5 supercell	4	75	8,640	448
MoS_2 25×25 supercell	4	1,875	3,520,800	5,632
MoS_2 35×35 supercell	4	3,675	22,704,120	10,688
(50, 0) MoS_2 nanotube	4	300	128,128	1,152

Supplementary Section 2. SUMMARY OF THE DATASET

Supplementary Table 2 summarizes the amount of data in each dataset. For graphene, the dataset containing 5000 configurations of graphene sampled at 300 K is divided into a training set, a validation set and a test set. For training and validation sets, the first 500 unstable frames are excluded, and one frame is taken out of every ten frames. In total, 450 configurations of graphene are taken out, with 270 configurations as the training set and 90 configurations as the validation set. The other 4640 configurations are used as the test set. Another 2000 configurations of graphene sampled in the *ab initio* molecular dynamics (AIMD) simulation with temperature increasing from 100 K to 400 K are also used to test the generalization capability of DeepH. For MoS_2, the first 500 unstable frames are excluded, and the remaining 500 frames are divided into a training set, a validation set and a test set, with 300, 100, and 100 structures of monolayer MoS_2 supercells, respectively. The neural networks are shared for different types of atom pairs and similar orbital combinations. In order to train the model for studying twisted bilayer graphene (TBG), 180, 60, and 60 structures from 4×4 random supercells of bilayer graphene with zero twist angle are used for training, validation, and test, respectively. For twisted bilayer bismuthene (TBB), 345, 115, and 115 structures from 3×3 random supercells of bilayer bismuthene with zero twist angle are used for training, validation, and test, respectively.

Supplementary Table 2. The amount of data in each dataset and the data splitting used for training, validation, and test, respectively.

Data set	n_{data}	n_{training}	$n_{\mathrm{validation}}$	n_{test}
Graphene (300 K)	5,000	270	90	4,640
Graphene (100 K – 400 K)	2,000	/	/	/
MoS_2	500	300	100	100
Bilayer graphene	300	180	60	60
Bilayer bismuthene	576	345	115	115

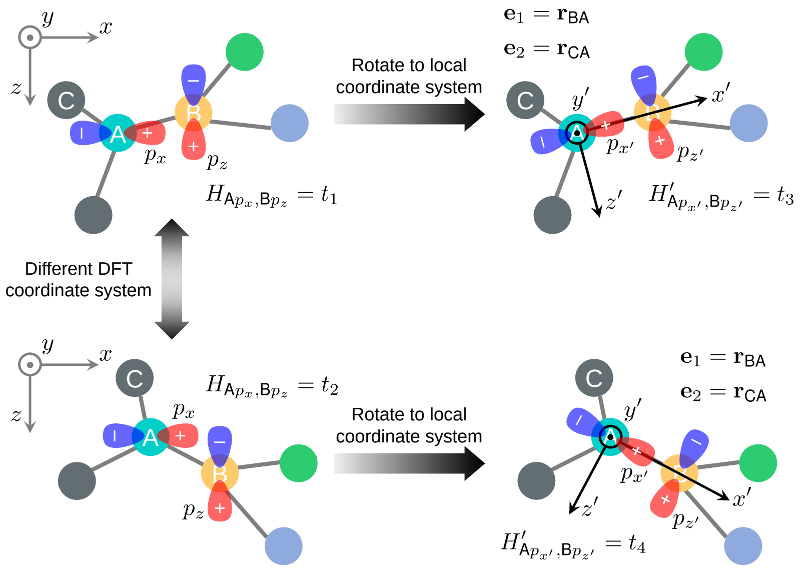

Supplementary Figure 1. Illustration of global and local coordinate systems. Structures in (global) DFT (xyz) and local ($x'y'z'$) coordinates are shown on the left and right, respectively. For the hopping term between orbital p_x of atom A and orbital p_z of atom B, when the material is rotated to a different DFT coordinate system, the hopping term in DFT coordinate system is covariant, i.e., t_1 can be transformed into t_2 using Eq. (5). In the local coordinate system, which is unique for every bond, x' is always alone $\mathbf{r}_{\mathrm{BA}}$, and y' is alone the cross product of $\mathbf{r}_{\mathrm{BA}}$ and $\mathbf{r}_{\mathrm{CA}}$, where C is the nearest neighbor of atom A. Because the local coordinate system rotates simultaneously with the material, hopping term in local coordinate system is invariant, i.e., $t_3 = t_4$.

Supplementary Section 3. LOCAL COORDINATE AND GAUGE TRANSFORMATION

Supplementary Section 3.1. Gauge transformation

Unlike physically measurable quantities, Hamiltonian matrix, which depends on the choice of basis functions (i.e. $\tilde{H} = U^\dagger H U$), is gauge covariant but not invariant. For simplicity we choose real-valued orbital functions as basis to partially fix the gauge. After that, the remaining freedom is the rotation of coordinate or change of basis functions.

Global rotation is a special case of local rotation and can be viewed as basis transformation. If we fix the basis (coordinate) but rotate the structure, or *vice versa*, the Hamiltonian matrix is covariant under rotations. However, if we choose the basis to rotate with the structure simultaneously, the Hamiltonian matrix is invariant under rotations. Special attention need to be paid to the local rotation of orbital functions. Different Hamiltonian matrix blocks $H'_{ij}(\{\mathcal{R}\}_N)$ learned by neural network in their local coordinate systems cannot be assembled directly. To construct the entire Hamiltonian matrix, an inverse orbital rotation needs to be applied that changes basis functions from their local coordinate system back to the original one used in training DFT calculations. More details about the local coordinate are discussed below.

Supplementary Section 3.2. Definition of local coordinate

Before training, the Hamiltonian matrix block $H_{i\alpha j\beta}$ is rotated to its local coordinate system defined by two vectors $\mathbf{e}_1$ and $\mathbf{e}_2$ that are determined by local chemical environment. For bonds of the A atom, we sort the neighboring atoms k by distance $|\mathbf{r}_{k\mathrm{A}}| = |\mathbf{r}_k - \mathbf{r}_\mathrm{A}|$ and define an ordered sequence $\{\mathbf{r}_{k\mathrm{A}} | k \in \mathcal{N}_\mathrm{A}, |\mathbf{r}_{k\mathrm{A}}| \leqslant |\mathbf{r}_{k+1,\mathrm{A}}|\}$. For Hamiltonian matrix block H_{AA} ($i = j = \mathrm{A}$), the two vectors are defined as

$$\mathbf{e}_1 = \mathbf{r}_{1\mathrm{A}}, \mathbf{e}_2 = \mathbf{r}_{\mu\mathrm{A}} \tag{1}$$

with μ being the lowest number satisfying $\mathbf{r}_{\mu\mathrm{A}} \times \mathbf{r}_{1\mathrm{A}} \neq 0$. For Hamiltonian matrix block H_{AB} ($i = \mathrm{A}, j = \mathrm{B}$) with $\mathrm{A} \neq \mathrm{B}$, the two vectors are defined as (Supplementary Figure 1)

$$\mathbf{e}_1 = \mathbf{r}_{\mathrm{BA}}, \mathbf{e}_2 = \mathbf{r}_{\nu\mathrm{A}} \tag{2}$$

with ν being the lowest number satisfied $\mathbf{r}_{\nu\mathrm{A}} \times \mathbf{r}_{\mathrm{BA}} \neq 0$. Accordingly, the local coordinate is defined as

$$\hat{x}' = \frac{\mathbf{e}_1}{|\mathbf{e}_1|}, \hat{y}' = \frac{\mathbf{e}_1 \times \mathbf{e}_2}{|\mathbf{e}_1 \times \mathbf{e}_2|}, \hat{z}' = \hat{x}' \times \hat{y}' . \tag{3}$$

The 3×3 rotation transformation between DFT coordinate and local coordinate associated with bond AB (include both B=A and B≠A) is simply given by

$$R^{\mathrm{AB}} = (\hat{x}', \hat{y}', \hat{z}') \tag{4}$$

with $\hat{x}', \hat{y}', \hat{z}'$ being the column vectors.

The Hamiltonian under local atomic basis is calculated in the following way:

1) Convert real spherical harmonics basis into complex spherical harmonics to make the rotation transformation below more convenient.

2) Apply rotation transformation to the Hamiltonian through

$$H'_{\mathrm{A}\alpha,\mathrm{B}\beta} = \sum_{a,b} D^{l_\alpha}_{\alpha,a} \left(R^{\mathrm{AB}}\right) H_{\mathrm{A}a,\mathrm{B}b} D^{l_\beta}_{b,\beta} \left(\left(R^{\mathrm{AB}}\right)^{-1}\right), \tag{5}$$

   where $D^{(l)}$ is the Wigner matrix, and l_α is the azimuthal quantum number.

3) Convert back to real spherical harmonics basis. With time reversal symmetry and $SU(2)$ spin rotational symmetry, the Hamiltonian matrix under this basis will be pure real, which will be computationally efficient for deep learning.

The predicted $H'_{i\alpha,j\beta}$ cannot be used to directly construct the full Hamiltonian matrix. Instead, we need to change back to DFT coordinates by

$$H_{\mathrm{A}\alpha,\mathrm{B}\beta} = \sum_{a,b} D^{l_\alpha}_{\alpha,a} \left(\left(R^{\mathrm{AB}}\right)^{-1}\right) H'_{\mathrm{A}a,\mathrm{B}b} D^{l_\beta}_{b,\beta} \left(R^{\mathrm{AB}}\right). \tag{6}$$

With the above techniques, our method naturally preserves the rotation covariance of DFT Hamiltonian matrix.

Supplementary Section 3.3. Importance of local coordinate message passing (LCMP) Layer

Although features in our graph including atomic number Z_i and interatomic distance $|r_{ij}|$ already contain enough information to predict rotation-invariant properties, orientation information of local coordinate systems is important to deep learning. The local coordinate system defined in Supplementary Section 3.2 does not change continuously with varying interatomic distance. Thus the resulting Hamiltonian matrix H' may change abruptly under minor structural perturbation, as illustrated in Supplementary Figure 2. The upper and lower structures in Supplementary Figure 2 are differed by a small variation of interatomic distance. However, y'-axis and z'-axis of local coordinate are considerably different for the two structures. As a result, the Hamiltonian matrix element between orbital p_x of atom A and orbital p_z of atom B are opposite for the two structures, i.e., $t_1 \approx -t_2 \neq t_2$. If only the interatomic distance is included in the edge feature, input features of the two structures are similar $\mathcal{G}_1 \approx \mathcal{G}_2$, and it cannot result in an abrupt change of output from deep neural network. Therefore, it is essential to include the LCMP layer, which contains orientation information of bonds relative to local coordinate as edge features.

The general formulae of introducing LCMP layers after the M-th message passing (MP) layer are given by

$$v_i^{pq(M+1)} = \sum_{k\in\mathcal{N}_i} \Phi_v^{(M+1)} \left(z_{ik}^{(M)} \parallel \{Y_{Jm}(\theta_{ik}^{pq}, \phi_{ik}^{pq})\}\right), \tag{7}$$

$$e_{ij}^{pq(M+1)} = \Phi_e^{pq(M+1)} \left(v_i^{(M+1)} \parallel v_j^{pq(M+1)} \parallel e_{ij}^{(M)}\right), \tag{8}$$

$$v_i^{pq(M+l)} = \sum_{k\in\mathcal{N}_i} \Phi_v^{(M+l)} \left(z_{ik}^{pq(M+l-1)}\right), \tag{9}$$

$$e_{ij}^{pq(M+l)} = \Phi_e^{(M+l)} \left(v_i^{pq(l)} \parallel v_j^{pq(M+l)} \parallel e_{ij}^{pq(M+l-1)}\right). \tag{10}$$

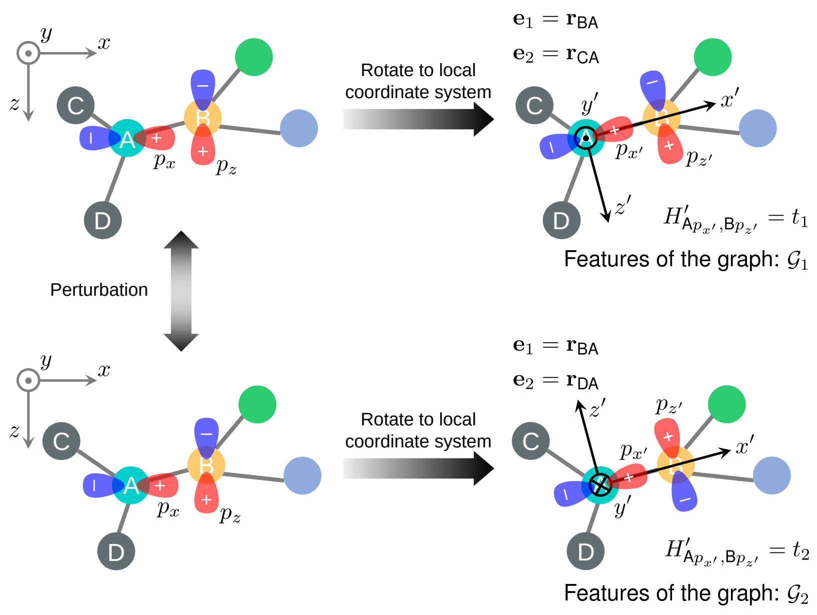

Supplementary Figure 2. Discontinuous change of local coordinate under minor structural perturbation. Thus it is essential to include the orientation information of bonds relative to local coordinate system in the input of deep learning method.

Eqs. (7) and (8) describe the first LCMP layer, whereas Eqs. (9) and (10) describe the l-th LCMP layer ($l \geq 2$). θ_{ik}^{pq} and ϕ_{ik}^{pq} are the corresponding polar and azimuthal angles, respectively, of bond ik relative to a local coordinate defined for edge pq. A set of real spherical harmonic functions $\{Y_{Jm}\}$ are used to capture orientation information.

The computational cost of features with directional vectors in the local coordinate system is proportional to the square of the number of edges in a crystal graph. In order to improve efficiency, the interatomic distance without orientation is added to the edge feature in the first M layers of the MPNN model, followed by one single LCMP layer. Only vertex features at both ends of the bond ij and diagonal edge feature e_{ij}^{ij} need to be calculated to predict the Hamiltonian matrix element between atom pair i and j:

$$v_i^{ij(M+1)} = \sum_{k \in \mathcal{N}_i} \Phi_v^{(M+1)} \left(z_{ik}^{(M)} \parallel \left\{ Y_{Jm}(\theta_{ik}^{ij}, \phi_{ik}^{ij}) \right\} \right), \tag{11}$$

$$v_j^{ij(M+1)} = \sum_{k \in \mathcal{N}_j} \Phi_v^{(M+1)} \left(z_{jk}^{(M)} \parallel \left\{ Y_{Jm}(\theta_{jk}^{ij}, \phi_{jk}^{ij}) \right\} \right), \tag{12}$$

$$H'_{i\alpha,j\beta} = e_{ij}^{ij(M+1)} = \Phi_e^{(M+1)} \left(v_i^{ij(M+1)} \parallel v_j^{ij(M+1)} \parallel e_{ij}^{(M)} \right). \tag{13}$$

Supplementary Section 3.4. Ablation studies on the LCMP layer

In order to further demonstrate the importance of LCMP layer, we performed ablation studies on the LCMP layer and found that the removal of LCMP layer would greatly decrease accuracy. Specifically, we trained two models for Mo-Mo orbital pairs of monolayer MoS_2 dataset, one with the original architecture and the other replacing the final LCMP layer by a normal message passing layer. Then we computed the mean squared errors (MSE) of Hamiltonian matrix for the test set. Our experiment indicates that the removal of LCMP layer increases MSE by about three orders of magnitudes (Supplementary Table 3).

Supplementary Table 3. Results for ablation studies on the LCMP layer.

	Test set MAE (meV)	Test set MSE (eV^2)
Baseline	0.8	0.000004
No LCMP layer	16.8	0.007931

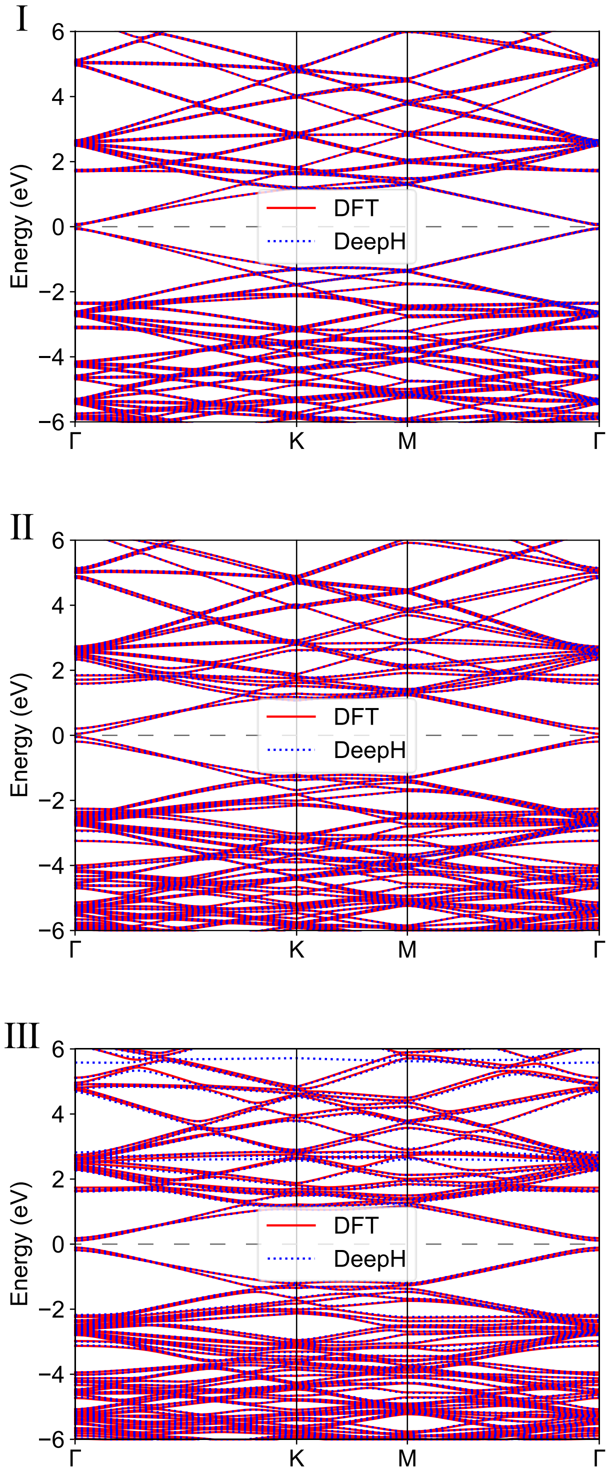

Supplementary Figure 3. Performance of DeepH on studying monolayer graphene. Band structures of the three representative graphene supercells with the best, median, and the worst mean absolute errors of DOS, respectively, for dataset sampled by AIMD from 100 K to 400 K.

Supplementary Section 4. DETAILED RESULTS OF EXAMPLE MATERIALS STUDIES

Supplementary Section 4.1. Monolayer graphene

Three representative structures of distorted graphene supercells, whose calculated properties are shown in Fig. 3, are included in Supplementary Data 1. Supplementary Figure 3 shows the comparisons of band structures for these three structures. Some part of results are shown in Fig. 5b in the main text.

In order to demonstrate the convergence of the learning curve as a function of training set size, neural networks are trained to represent the Hamiltonian matrix element $H'_{i1,j1}$ by using varying numbers of training structures and the mean absolute error (MAE) of $H'_{i1,j1}$ for 300 test structures are calculated. The results are summarized in Supplementary Figure 4. As the size of training set increases, the MAE of $H'_{i1,j1}$ first reduces significantly, then fluctuates moderately due to randomness of parameter initialization and training process, and finally approaches a converged value. 270 structures were used for training in our original work which is large enough to ensure convergence. Note that each supercell structure contains thousands of nonzero Hamiltonian matrix blocks and millions of data can be provided by hundreds of training structures in our example study. The large amount of data provided by a few training structures enables accurate and efficient learning of DFT Hamiltonian.

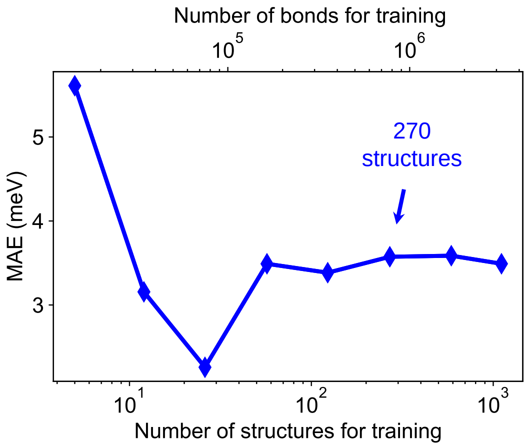

Supplementary Figure 4. Learning curve as a function of training set size on studying monolayer graphene. MAE of $H'_{i1,j1}$ for varying number of structures (or bonds) for training, as calculated for 300 test structures of monolayer graphene supercell.

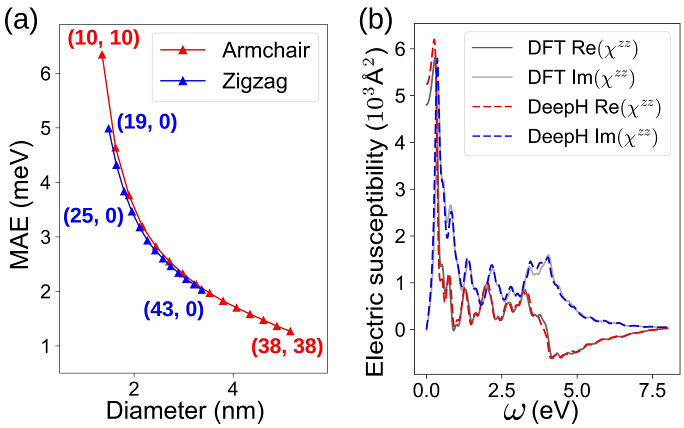

Supplementary Figure 5. Generalization ability of DeepH on CNTs. (a) Averaged MAE among 169 orbital pairs of $H'_{i\alpha,j\beta}$ for armchair and zigzag CNTs with varying diameters. (b) Real and imaginary parts of electric susceptibility χ^{zz} for a zigzag (25, 0) CNT computed by DFT and DeepH. The periodic direction of CNT is defined as the z-axis.

Supplementary Section 4.2. Carbon nanotubes (CNTs)

We test the performance of models training on monolayer graphene dataset by making predictions on CNTs (unseen in the training set), which have curved quasi-one-dimensional atomic structures, significantly distinct from graphene. 15 armchair and 13 zigzag CNTs with different chiral indexes are used to test the transferability of the DeepH method. The chiral index armchair CNTs ranges from (10, 10) with diameter ≈ 1.4 nm to (38, 38) with diameter ≈ 5.2 nm. The chiral index of zigzag CNTs range from (19, 0) with diameter ≈ 1.5 nm to (43, 0) with diameter ≈ 3.4 nm.Overall, the averaged MAE of DFT Hamiltonian matrix is insensitive to nanotube chirality and monotonically decreases with increasing nanotube diameter (d), which reduces to below 3.5 meV for $d > 2$ nm (Supplementary Figure 5(a)). For a zigzag $(25, 0)$ CNT ($d \sim 2$ nm), the predicted band structure (Fig. 5(b) in the main text) and electric susceptibility as a function of frequency (Supplementary Figure 5(b), corresponding to inter-band transitions) can well reproduce DFT calculation results. Remarkably, properties of large-diameter CNTs can be accurately predicted by DeepH method at very low computational expense.

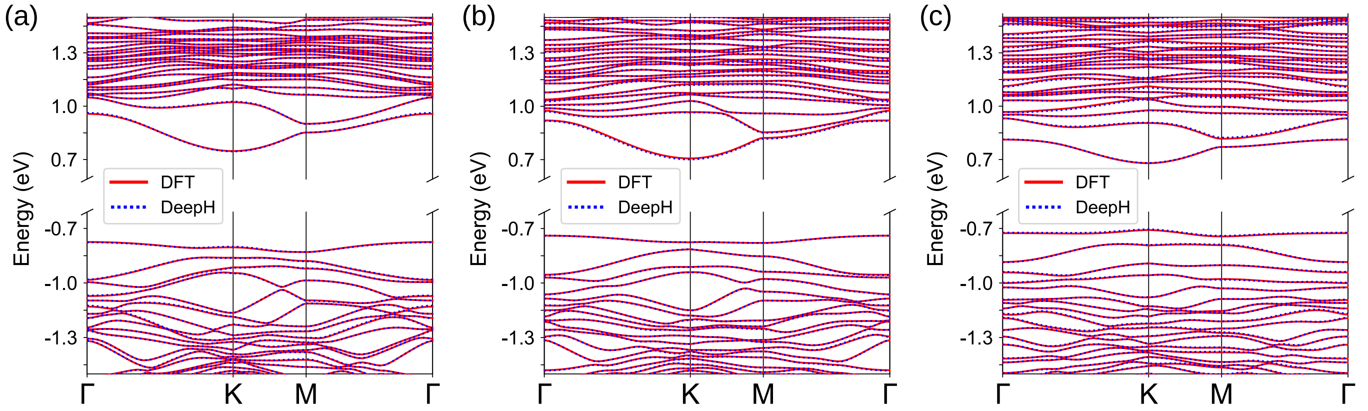

Supplementary Figure 6. Performance of DeepH on studying monolayer MoS_2. Band structure of three representative MoS_2 supercells with (a) the best, (b) median (shown in Fig. 4 (c) in the main text), and (c) the worst averaged MAE of Hamiltonian matrix elements among 100 structure in the test set, respectively, computed by DFT (red line) and DeepH (blue dot).

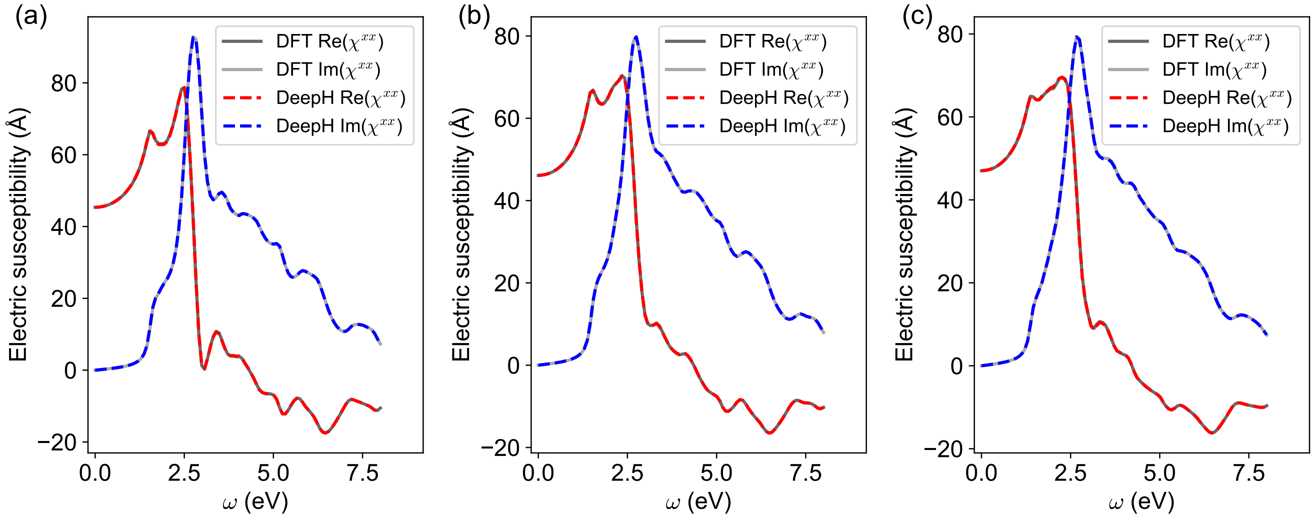

Supplementary Figure 7. Performance of DeepH on studying monolayer MoS_2. Real and imaginary parts of electric susceptibility χ^{xx} of three representative MoS_2 supercells with (a) the best, (b) median (shown in Fig. 4 (d) in the main text), and (c) the worst averaged MAE of Hamiltonian matrix elements among 100 structure in the test set, respectively, computed by DFT and DeepH.

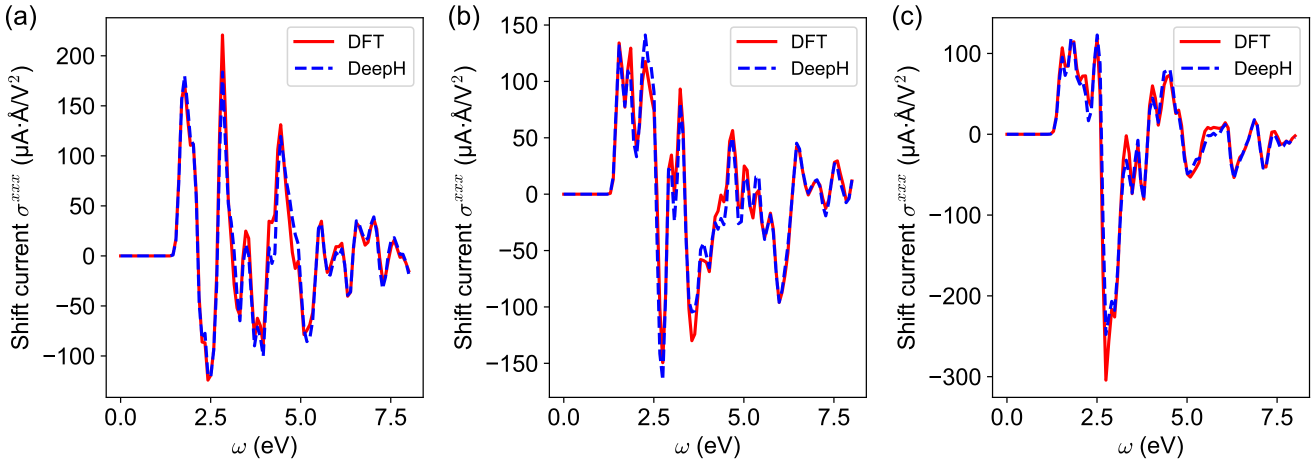

Supplementary Figure 8. Performance of DeepH on studying monolayer MoS_2. Shift current conductivity σ^{xxx} of three representative MoS_2 supercells with (a) the best, (b) median, and (c) the worst averaged MAE of Hamiltonian matrix elements among 100 structure in the test set, respectively, computed by DFT and DeepH.

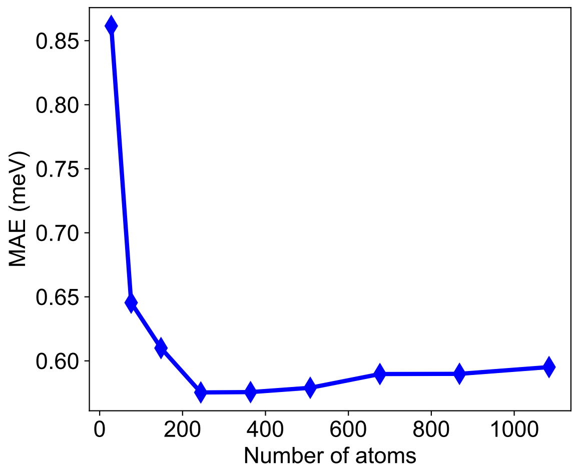

Supplementary Figure 9. Performance of DeepH on studying TBG. The averaged MAE among 169 orbital pairs of $H'_{i\alpha,j\beta}$ between the DFT calculated and the predicted values for TBGs of varying numbers of atoms.

Supplementary Section 4.3. Monolayer MoS_2

Band structures, electric susceptibility χ^{xx} and shift current conductivity σ^{xxx} are compared for three representative MoS_2 supercells with the best, median and worst averaged MAE of Hamiltonian matrix elements in Supplementary Figures 6, 7 and 8. Atomic structures of these supercells are included in Supplementary Data 2. Some part of results are shown in Fig. 5c in the main text.

Supplementary Section 4.4. Twisted bilayer graphene (TBG)

Supplementary Figure 9 shows the averaged MAE of $H'_{i\alpha,j\beta}$ between the DFT calculated and the predicted values for TBGs of varying numbers of atoms. Supplementary Figure 10 shows the comparisons of band structures for TBGs of varying twist angles. Part of the results are shown in the main text.

In addition to the comparison of band structures, the uncertainty of an ensemble of neural networks can also serve as a reliability indicator of accuracy for the TBG at the magic angle, which is widely used in accuracy estimation

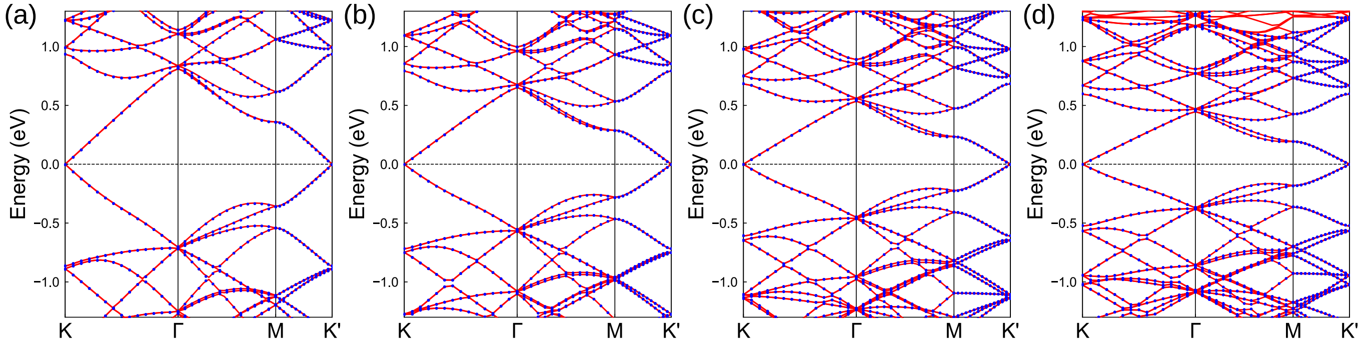

Supplementary Figure 10. Performance of DeepH on studying TBG. Band structures of TBGs of varying twist angles: (a) $\theta \approx 6.01°$ (364 atoms per supercell), (b) $\theta \approx 5.09°$ (508 atoms per supercell), (c) $\theta \approx 4.41°$ (676 atoms per supercell), (d) $\theta \approx 3.89°$ (868 atoms per supercell) computed by DFT (red) and DeepH (blue). The Fermi level is aligned according to the DFT results.

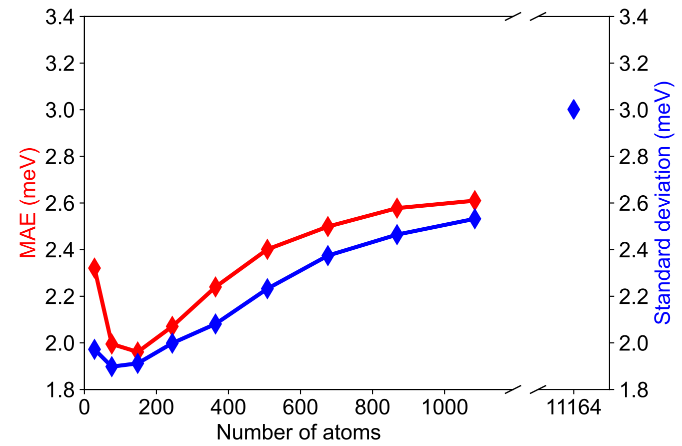

Supplementary Figure 11. Uncertainty of an ensemble of neural networks for TBG. Standard deviation (blue) of the predictions obtained from 10 different neural networks averaged over graph edges and the MAE (red) of $H'_{i6,j6}$ between the DFT calculated and the mean of predicted values for TBGs of varying numbers of atoms.

for complex material systems. As an example, 10 neural network models are trained to represent the Hamiltonian matrix element $H'_{i6,j6}$ of bilayer graphene supercells by using different random seeds and are used to predict $H'_{i6,j6}$ of TBGs of varying twist angle. The results are summarized in Supplementary Figure 11 and a very similar trend of the standard deviation of an ensemble of neural networks and the MAE are observed, which supports that the uncertainty of an ensemble of neural networks can serve as a reliability indicator. For the TBG at the magic angle $\theta \approx 1.08°$ with 11164 atoms, while it is almost not feasible to calculate the DFT Hamiltonian by self-consistent field iterations, the low standard deviation indicates a low MAE and good accuracy of our predictions.

Supplementary Section 4.5. Twisted bilayer bismuthene (TBB)

Band structures of AA stacking bilayer bismuthene computed by Vienna *ab initio* simulation package (VASP) and OpenMX package are compared in Supplementary Figure 12, illustrating the reliability of the training set. All the calculation of TBBs is preformed with spin-orbit coupling (SOC). Supplementary Figure 13 shows the averaged MAE of $H'_{i\alpha,j\beta}$ between the DFT calculated and the predicted values for TBBs of varying numbers of atoms. MAEs of the real and imaginary parts of the entire Hamiltonian with SOC are plotted. Supplementary Figure 14 shows the comparisons of band structures for TBBs of varying twist angles. Part of the results are shown in the main text.

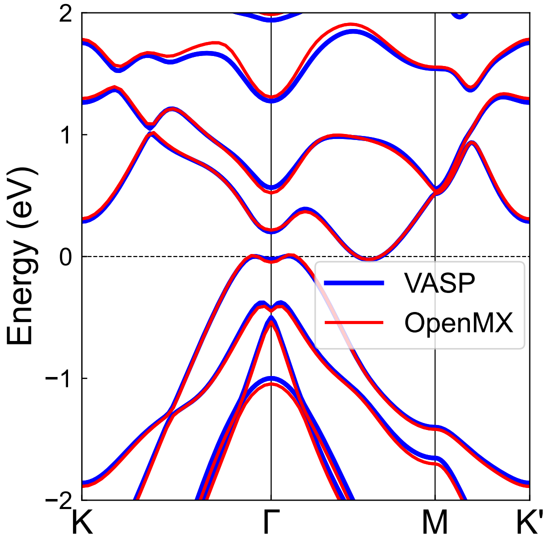

Supplementary Figure 12. Band structures of AA stacking bilayer bismuthene computed by VASP and OpenMX package.

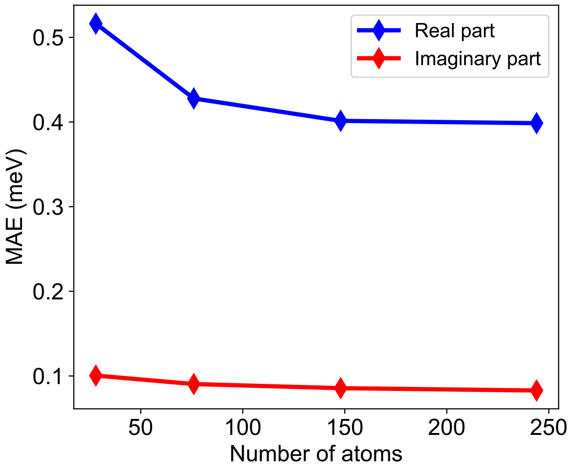

Supplementary Figure 13. Performance of DeepH on studying TBB. The averaged MAE among 361 orbital pairs of $H'_{i\alpha,j\beta}$ between the DFT calculated and the predicted values for TBBs of varying numbers of atoms. The real part and imaginary part for the entire Hamiltonian with spin-orbit coupling are plotted.

Supplementary Section 4.6. Bulk silicon

We used DeepH to study three-dimensional structures of bulk silicon. 300 4×4×4 bulk silicon supercells with random atomic displacements (up to 0.15 Å) with respect to equilibrium positions were prepared as dataset. Si7.0-$s2p1d1$ pseudo-atomic orbitals were used, including 10 atomic-like basis functions with the cut-off radius $R_C = 7.0$ Bohr. We used the trained model to make prediction on a 5×5×5 bulk silicon structure obtained by a 10 ps AIMD simulation at 600 K with the time step of 1 fs. The averaged MAE of Hamiltonian matrix is 2.0 meV, and the band structure predicted by DeepH matches well with that by DFT (Supplementary Figure 15).

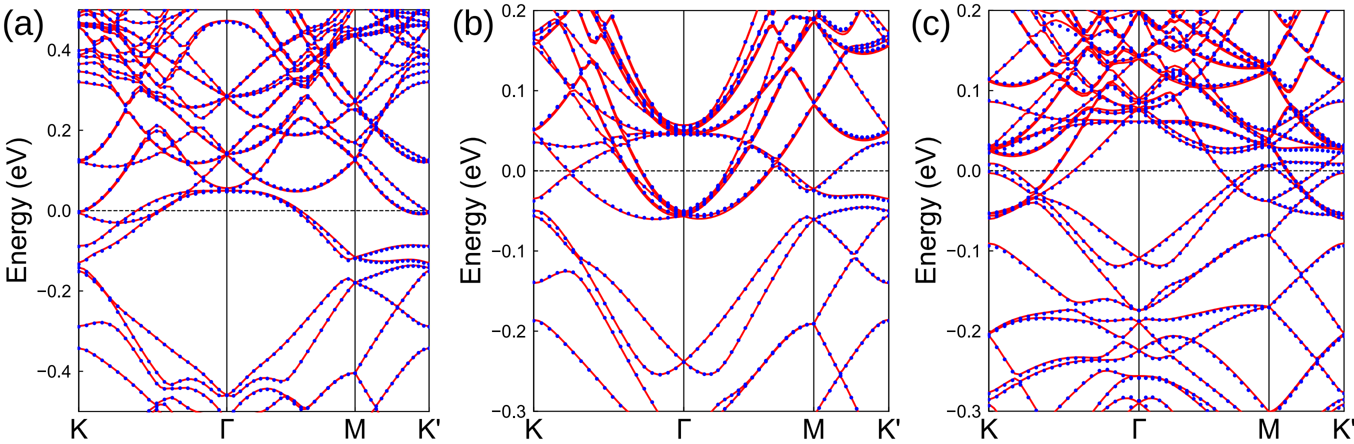

Supplementary Figure 14. Performance of DeepH on studying TBB. Band structures of TBBs of varying twist angles: (a) $\theta \approx 13.17°$ (76 atoms per supercell), (b) $\theta \approx 9.43°$ (148 atoms per supercell), (c) $\theta \approx 7.34°$ (244 atoms per supercell) computed by DFT (red) and DeepH (blue). The Fermi level is aligned according to the DFT results.

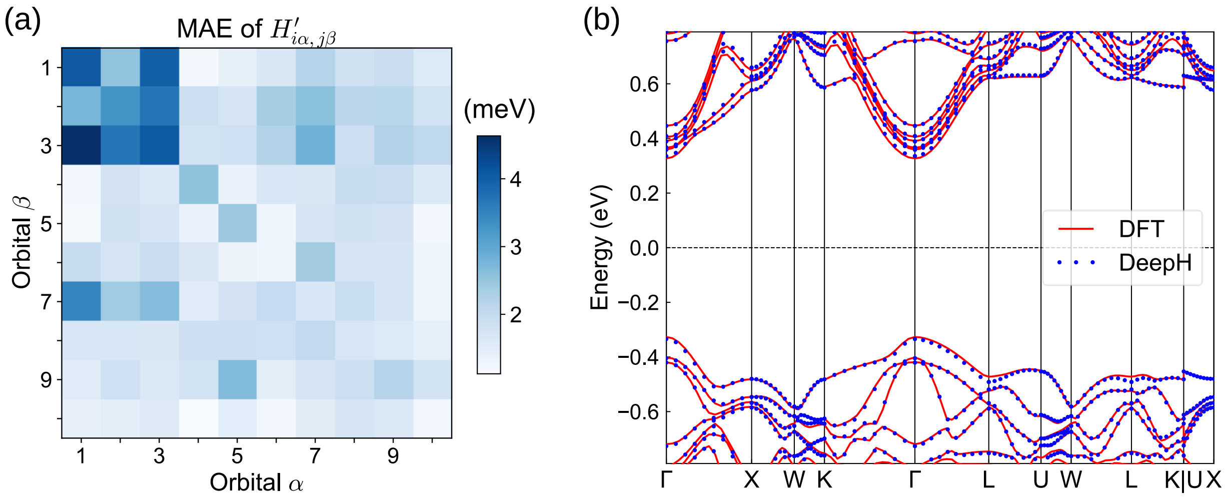

Supplementary Figure 15. Performance of DeepH on studying silicon. A 5×5×5 bulk silicon was obtained by the AIMD simulation, whose atomic structure is included in the Supplementary Data 3. (a) MAE of $H'_{i\alpha,j\beta}$ for different orbitals. (b) Band structure computed by DFT (red line) and DeepH (blue dot).

Supplementary Section 4.7. Solid structures with multiple crystalline phases

We trained a DeepH model using a dataset containing different allotropes of carbon, including graphite and diamond (Supplementary Figure 16). To construct such dataset, 300 3×3×3 graphite supercells and 300 3×3×3 diamond supercells with random atomic displacements (up to 0.15 Å) with respect to equilibrium positions are prepared. C6.0-$s2p2d1$ pseudo-atomic orbitals are used, including 13 atomic-like basis functions, with the cut-off radius $R_C = 6.0$ Bohr. A randomized training:validation:test split of 60:20:20 percent was used to test the quality of predictions. Here one unified neural network is applied to predict DFT Hamiltonian for two kinds of carbon allotropes. The MAEs of $H'_{i\alpha,j\beta}$ for graphite and diamond are shown in Supplementary Figure 16(b) and Supplementary Figure 16(d), respectively. The MAEs averaged over all the orbital combinations for graphite and diamond are as low as 1.50 and 2.04 meV, respectively.

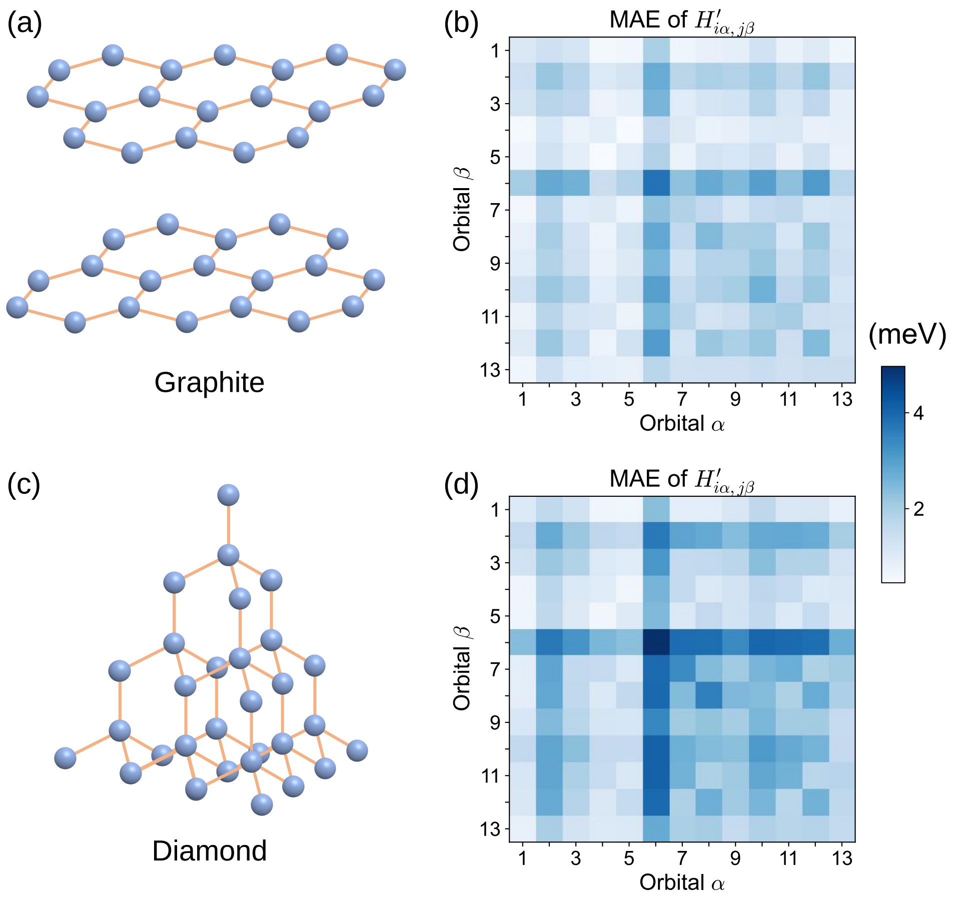

Supplementary Figure 16. Atomic structures (left panels) and mean absolute errors (MAEs) of $H'_{i\alpha,j\beta}$ for different orbitals (right panels) of (a, b) graphite and (c, d) diamond.

Supplementary Section 5. PRINCIPAL COMPONENT ANALYSIS (PCA)

Supplementary Section 5.1. PCA for nanotubes

We preformed PCA for the output atom features of the final message passing layer (i.e., input of the LCMP layer) on a random monolayer graphene supercell from training set and CNTs with different diameters (Supplementary Figure 17). 64-dimensional feature of each atom is mapped to one point in the PCA plot, which represents local structural information of atom. For the graphene supercell with randomly displaced geometry, there exist many kinds of local environments as indicated by the PCA plot. For CNTs, principal components (PCs) for different atoms in one CNT are localized at the same point due to structure symmetry. Here CNTs with larger diameters give larger PC1 value. Thus we guess that the value of PC1 to some extent reflects local curvature information of atomic structures. Remarkably, even for CNTs whose atomic features are far away from those of training data in the PCA plot, DeepH can well reproduce DFT calculation results for both Hamiltonian matrix elements (Supplementary Figure 5) and band structures (Supplementary Figure 17). This case study demonstrates the good generalization ability of DeepH, benefitting from the preservation of rotation covariance.

Similar as for CNTs, we also preformed PCA on one random monolayer MoS_2 from training set and a zigzag (50, 0) MoS_2 nanotube (Supplementary Figure 18). PCs for different atoms in the MoS_2 nanotube are localized at three points, which represent a Mo atom and two inequivalent S atoms. We guess that PC1 describes the element type, and PC2 describes the local structural stretching. Atomic features of MoS_2 nanotube are distinct from those of training data (monolayer MoS_2) in the PCA plot. The good prediction accuracy of DeepH on the MoS_2 nanotube demonstrates the generalization ability of DeepH.

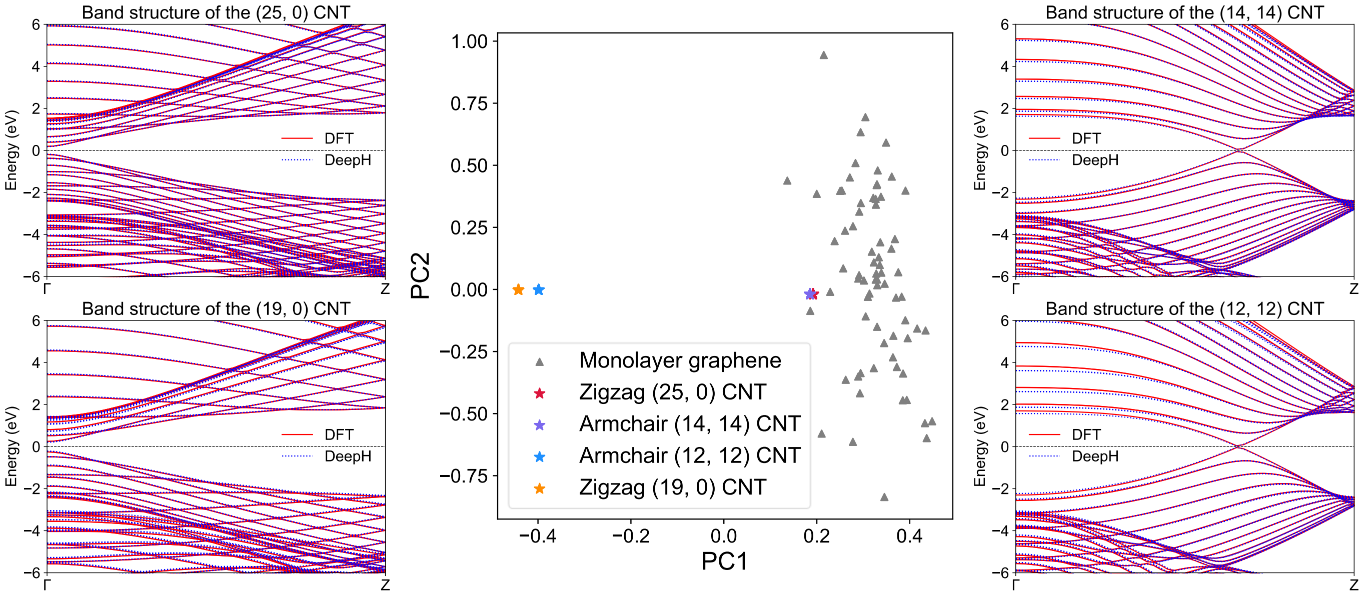

Supplementary Figure 17. PCA for graphene and CNT. PCA plot for atom features of the final LCMP layer, and the band structure comparisons by DFT and DeepH for CNTs whose PCs are far away from those of random monolayer graphene.

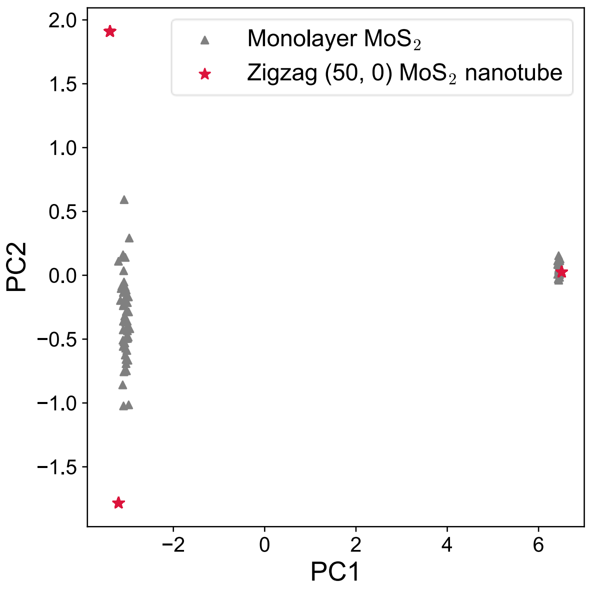

Supplementary Figure 18. PCA for monolayer MoS_2 and MoS_2 nanotube. PCA plot for atom features of the final LCMP layer on random monolayer MoS_2 supercell from training set and zigzag (50, 0) MoS_2 nanotube.

Supplementary Section 5.2. PCA for twisted bilayers

PCA plot for atom features on random bilayer graphene from training set and TBGs is displayed in Supplementary Figure 19(a), which does not show important diversity of atom features for the two regular TBG systems. However, the situation changes if we perform PCA for bond features of the final LCMP layer, which is used to construct

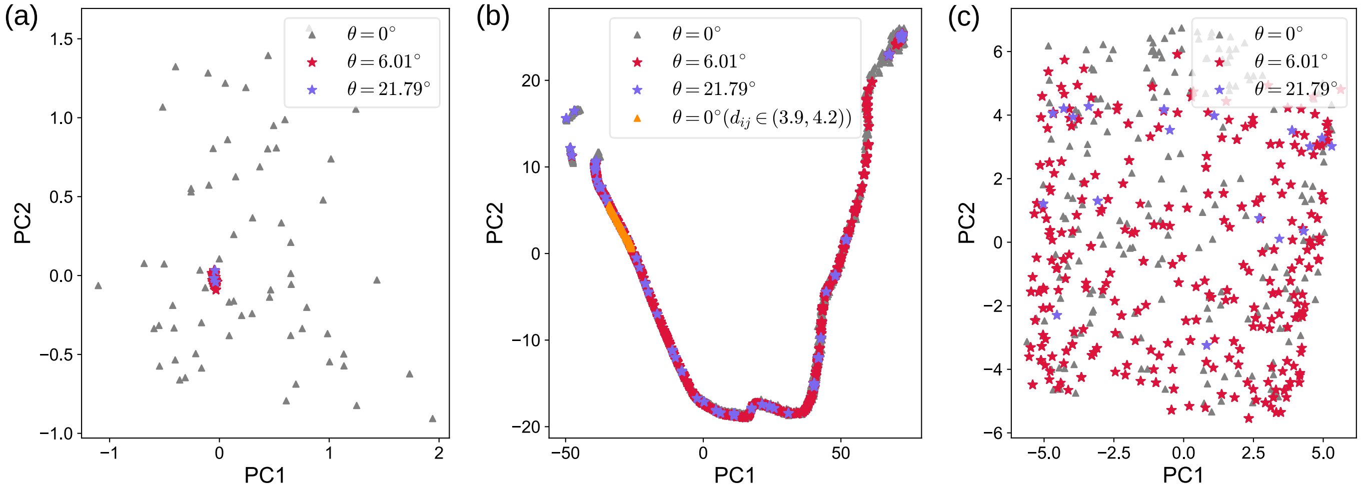

Supplementary Figure 19. PCA for TBG. PCA plot for (a) atom features of the final LCMP layer, (b) bond features of the final LCMP layer, and (c) bond features with atomic distance between 3.9 to 4.2 Å of the final LCMP layer on random bilayer graphene from training set ($\theta = 0°$) and TBGs ($\theta \neq 0°$).

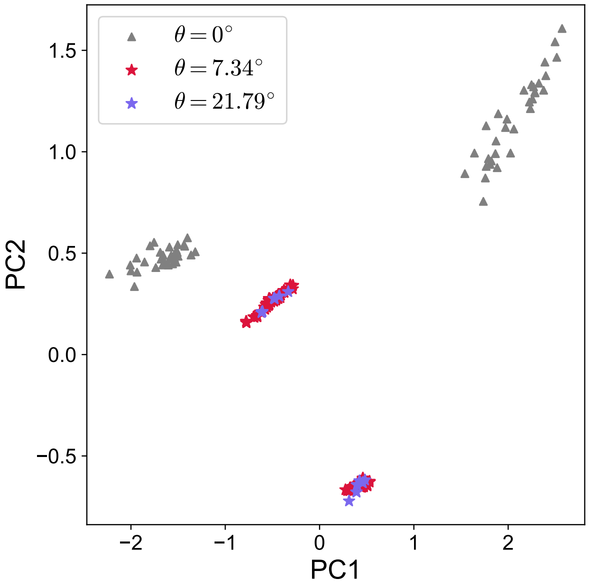

Supplementary Figure 20. PCA for TBB. PCA plot for atom features of the final LCMP layer on random bilayer bismuthene from training set ($\theta = 0°$) and TBBs ($\theta \neq 0°$).

Hamiltonian matrix blocks in neural networks. Bonds features with atomic distances between 3.9 to 4.2 Å are localized in a small area (orange points in Supplementary Figure 19(b)), indicating that PCs for bond features mainly describe the interatomic distance. Furthermore, we performed PCA for bond features focusing on the atomic distance interval between 3.9 to 4.2 Å. Then you may find that the PCs of bond features on TBGs are different from those of training data (Supplementary Figure 19(c)), illustrating the generalization ability of DeepH for studying varying bonding environments.

PCA for atom features of the final LCMP layer on twisted bilayer bismuthenes (TBBs) is different from that on TBGs (Supplementary Figure 20). PCs on random bilayer bismuthene from training set or TBBs are localized in two small areas in the PCA plot, possibly corresponding to the surface and subsurface atoms. The PCs on the corresponding two areas for random bilayer bismuthene with zero twist angle (training set) are both far away from PCs for TBBs.

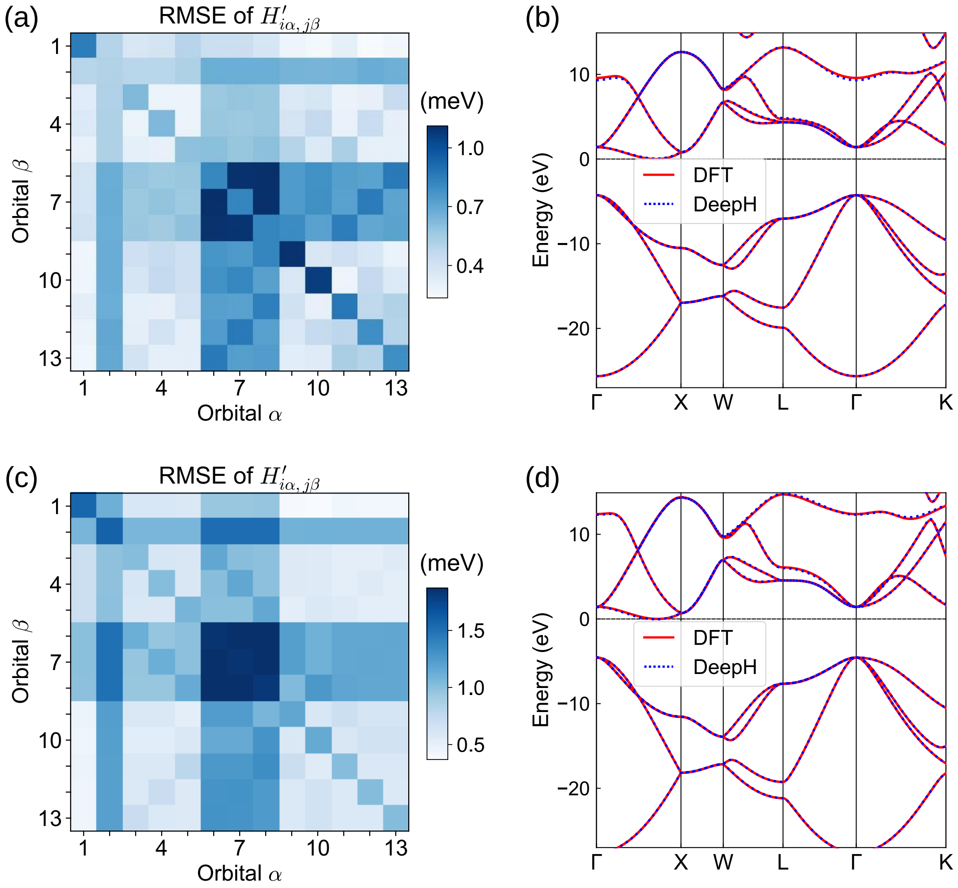

Supplementary Figure 21. Performance of DeepH on studying (a, b) unstrained and (c, d) 4% strained C-diamond. (a, c) Averaged root mean square error (RMSE) of $H'_{i\alpha,j\beta}$ for different orbitals. (b, d) Band structures computed by DFT and DeepH.

Supplementary Section 6. COMPARISON WITH OTHER METHODS

Supplementary Section 6.1. Comparison with the kernel ridge regression method

Ref. [1] learned the DFT Hamiltonian matrix by the kernel ridge regression (KRR) method for strained diamond system. Here we performed experiments to make quantitative comparisons with the referenced KRR method. Specifically, we trained a DeepH model using similar dataset as the reference (i.e., 40 unit-cell diamond systems with random lattice strains within 4%), and used DeepH to make prediction on unstrained and strained diamond structures. C6.0-$s2p2d1$ pseudo-atomic orbitals were used, including 13 atomic-like basis functions, with the cut-off radius $R_C = 6.0$ Bohr. DeepH shows high prediction accuracy for both DFT Hamiltonian matrix and band structure (Supplementary Figure 21). On the unstrained diamond, the averaged root mean square error (RMSE) of the predicted DFT Hamiltonian matrix elements is 0.6 meV, and the highest RMSE of p-p orbital coupling is 1.1 meV. In contrast, the p-p RMSE obtained by the KRR method is about 50 meV in the reference. Moreover, band structure of DFT can be well reproduced by DeepH, whereas the results of KRR show significant deviations in unoccupied bands ([1]). It is thus concluded that DeepH outperforms the KRR method even for the study of simple material structures.

Supplementary Section 6.2. Comparison with SchNOrb and PhiSNet

PhiSNet ([2]) applied a tensor-product-based method to predict DFT Hamiltonian matrix for small molecules

Supplementary Table 4. Comparison of performance with SchNOrb and PhiSNet for small molecule datasets.

Dataset	Inference time (s)			Number of parameters			Test set MAE (meV)		
	SchNorb	PhiSNet	DeepH	SchNorb	PhiSNet	DeepH	SchNorb	PhiSNet	DeepH
Water (H_2O, N_{train}: 500)	–	–	0.0005	$\sim 10^7$	$\sim 10^7$	$\sim 10^5$-10^6	4.501	0.479	1.048
Water (H_2O, N_{train}: 3000)	–	–	0.0005				–	–	0.593
Ethanol (C_2H_6O)	–	0.027	0.0023				5.099	0.331	0.601
Malondialdehyde ($C_3H_4O_2$)	–	0.029	0.0022				5.200	0.335	0.547
Uracil ($C_4H_4N_2O_2$)	–	0.050	0.0048				6.199	0.292	0.470
Aspirin ($C_9H_8O_4$)	–	0.155	0.0148				13.77	0.349	0.785

with fixed number of atoms. An earlier work (SchNOrb [3]) studied the similar problem, which did not apply covariant neural network but used data augmentation to satisfy rotation covariance requirement. To make quantitative comparisons with SchNOrb and PhiSNet, we considered the same molecular structures obtained by *ab initio* molecular dynamics as in these two references, performed the electronic structure calculations by the OpenMX code using H6.0-$s2p1$, C6.0-$s2p2d1$, N6.0-$s2p2d1$ and O6.0-$s2p2d1$ pseudo-atomic orbitals, and used the dataset to train, validate and test DeepH. Five kinds of molecules were studied, including water (H_2O), ethanol (C_2H_6O), malondialdehyde ($C_3H_4O_2$), uracil ($C_4H_4N_2O_2$), and aspirin ($C_9H_8O_4$). The same number of training data were used as PhiSNet if not explicitly mentioned. To compare the inference time, DeepH is evaluated on an NVIDIA RTX 3090 GPU with a batch size of 64. The comparisons of accuracy and efficiency are summarized in Supplementary Table 4.

Supplementary Section 7. COMPARISON OF THEORETICAL METHODS FOR TWISTED MATERIALS

Supplementary Table 5. Performance of different theoretical methods used to study twisted van der Waals materials.

Method	Accuracy	Speed	Choice of material	Choice of twist angle
Ab initio DFT	High	Low	High	Low
Empirical models	Low	High	Low	High
**DeepH**	**High**	**Medium-high**	**High**	**High**

[1] G. Hegde and R. C. Bowen, Machine-learned approximations to density functional theory hamiltonians, Sci. Rep. **7**, 42669 (2017).

[2] O. T. Unke, M. Bogojeski, M. Gastegger, M. Geiger, T. Smidt, and K.-R. Müller, Se(3)-equivariant prediction of molecular wavefunctions and electronic densities, in *Advances in Neural Information Processing Systems* (Curran Associates, Inc., 2021) p. 14434–14447.

[3] K. T. Schütt, M. Gastegger, A. Tkatchenko, K.-R. Müller, and R. J. Maurer, Unifying machine learning and quantum chemistry with a deep neural network for molecular wavefunctions, Nat. Commun. **10**, 5024 (2019).
